# A New Paradigm for Computational Chemistry

**DOI:** 10.1021/jacs.6c06653

**Published:** 2026-10-20

**Authors:** Raphael T. Husistein, Markus Reiher

**Affiliations:** Department of Chemistry and Applied Biosciences, 31064 ETH Zurich, Vladimir-Prelog-Weg 2, Zurich 8093, Switzerland

## Abstract

Computational chemistry has become an indispensable tool for generating data and insights, pervading all branches of experimental chemistry. Its most central concept is the potential energy hypersurface, which is key to all chemistry and materials science, as it assigns an energy to a molecular structure, the necessary ingredient for reaction mechanism elucidation and reaction rate calculation. Density functional theory (DFT) has been the most important method in practice for obtaining such energies, which is reflected in the use of high-performance computing hardware. In the last two decades, a new class of surrogate potential energy functions has evolved with remarkable properties: quantum accuracy combined with force-field speed. Until very recently, their application was hampered by the fact that they needed to be trained on truly large system-specific data sets, generated before a computational chemistry study could be started (in sharp contrast to DFT, which, as a first-principles method, works out of the box but at a far higher price of computational cost). Very recently, this roadblock has been overcome by so-called foundation machine learning interatomic potentials, which are poised to completely change the way we do computational chemistry, likely prompting us to abandon DFT as the prime method of choice for this purpose in less than a decade.

## Graphical abstract

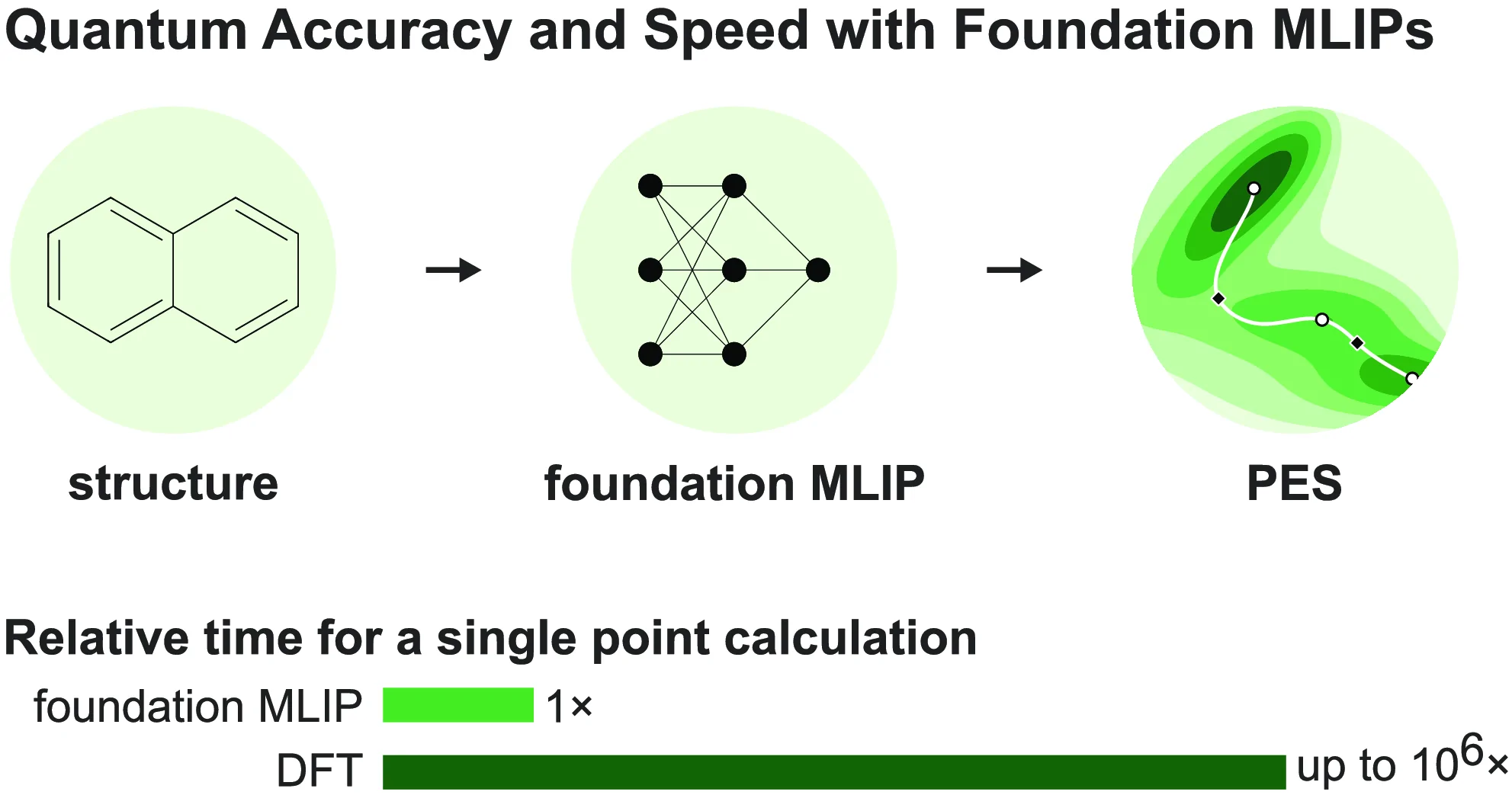

## 1 Introduction

Central to our understanding of chemical processes is the assignment of a potential energy to a molecular structure, more specifically to a nuclear configuration, which leads to the concept of a potential energy surface that allows us to study reaction chemistry in terms of stable intermediates, reaction paths, transition-state structures, and so forth. It is the Born–Oppenheimer approximation, the so-called clamped-nuclei approximation, which allows us to calculate the electronic energy of such a fixed arrangement of atomic nuclei. There exists a plethora of quantum chemical methods to calculate this electronic energy, all characterized by some balance of accuracy and feasibility. These methods allow us to predict reaction energies and activation barriers routinely,^1,2^ alongside molecular structures and transition states on the basis of the first-principles of quantum mechanics, an accomplishment that was recognized by a share of the 1998 Nobel Prize in Chemistry to John Pople.

In the 1990s, approximate density functional theory (DFT) took the lead for this purpose within computational chemistry owing to its convincing compromise of bearable computational cost and sufficient reliability, leading to the recognition of its impact on chemistry by the other half of the 1998 Nobel Prize in Chemistry to Walter Kohn. More than 90% of all quantum chemistry calculations are of DFT type^3^ and about one-third of all computer science applications on supercomputer systems.^4^ Hence, the practical and economic importance, as well as the environmental footprint of DFT, can hardly be overestimated.

The accuracy (and to some degree the efficiency) of DFT depends on the approximate form chosen for the so-called exchange–correlation (xc) density functional, typically denoted by one of the notorious capital-letter acronyms. Although an exact xc functional can be shown to exist, its analytical form is not known and would probably also be too involved for efficient computational chemistry approaches. Hence, rather drastic and often heavily parametrized approximations are adopted in practice, while attempts are made to fulfill the comparatively small number of known rigorous constraints. However, the approximate potentials, which are obtained from these approximate xc energy functionals as functional derivatives, differ significantly from reconstructed reference potentials.^5–7^ The fact that such xc functionals nevertheless produce useful results in chemical applications can be taken as an indication that there must be some sort of error compensation present. In fact, error compensation can be exposed if approximate exchange functionals are replaced by exact local exchange in Kohn–Sham DFT calculations, which then no longer compensates for deficiencies in the approximation to the correlation functional.^8^

As a result, practical DFT calculations have a significant semiempirical character. An important consequence is that they cannot be systematically improved. However, it would already be sufficient to know the system-focused error of some specific DFT calculation,^9^ but rigorous uncertainty quantification is not easy (see, for instance, ref 10) and Bayesian approaches have their limits.^11,12^ Usually, one acquires empirical knowledge about the reliability of DFT results over time, by which one can establish some ’gut feeling’ regarding the reliability of certain xc functionals in specific calculations. We emphasize this touch of an anthropogenic bias to prepare the ground for a higher acceptance of the paradigm shift brought about by machine learning inference models.

The last two decades have seen an intense development of what is now called machine learning interatomic potentials (MLIPs).^13,14^ In their seminal paper from 2007, Behler and Parrinello^15^ proposed high-dimensional neural network potentials that were applicable to large atomistic systems (by virtue of an architecture that scales linearly in the number of atoms), yielded accurate results on par with DFT (which was used as reference for their system-focused training), and obeyed the proper symmetries. The value of their approach became clear quickly, because these neural network potentials were able to deliver the accuracy of quantum chemical methods combined with the computational efficiency of classical force fields. However, they came with a huge burden regarding their parametrization. Thousands of quantum calculations were needed to parametrize such MLIPs, which created a huge barrier for general-purpose applications—but this situation has changed in the past few years. Foundation models were developed that demonstrate remarkable extrapolation capabilities, so that initial pretraining is no longer mandatory.^16,17^ Even chemical reactions can now be studied out of the box for many systems. The tremendous success of these approaches makes them the natural enemy of the current canonical choice for computational chemistry applications, that is, DFT.

In this work, we provide a brief review of MLIPs for the approximation of potential energy surfaces. We discuss their strengths and current weaknesses in order to eventually paint a deliberately forward-looking and opinionated picture of future computational chemistry, relying on foundation MLIPs as the entry point for calculations in molecules and materials.

## 2 Machine Learning Interatomic Potentials

We first review and discuss some basic theoretical foundations of this new powerful class of potential energy functions. Various names have been used in the literature that all refer to the same concept: machine learning potential (MLP), machine learning force field (MLFF), and machine learning interatomic potential (MLIP). Since MLP could be mistaken for “multilayer perceptron,” there has been a shift toward the term machine learning interatomic potential and the corresponding abbreviation “MLIP.” MLIPs can be developed using various machine learning algorithms, such as linear regression, Gaussian process regression, and neural networks. Recently, however, MLIPs based on neural networks have become predominant in the field, so we will focus on those.

MLIPs must meet two requirements in order to describe atomic interactions effectively and accurately. First, they need to encode a molecule or atomistic assembly in a way that enables machine learning algorithms that rely on fixed-size inputs to work with systems of arbitrary size. Second, MLIPs must address the physical symmetries inherent to the problem. Specifically, the predictions of the energy should remain invariant under translations and rotations of the entire atomistic system and when identical atoms are swapped. The first requirement is typically met by expressing the total energy as a sum of local (atomic) contributions,

1 $$E_{\mathrm{tot}}=\sum_{i=1}^{N}E_{i}\left(\{r_{j},Z_{j}|∥r_{j}-r_{i}∥<r_{c}\}\right)$$

where *N* is the total number of atoms in the system, and each atomic contribution *E*_*i*_ depends both on the position **r**_*j*_ and the chemical element expressed in terms of the nuclear charge number *Z*_*j*_ of all neighboring atoms *j* within a cutoff radius *r*_c_ of atom *i*. Physically, this approach can be motivated by the “nearsightedness of electronic matter”.^18^ However, this assumption of locality implies that long-range interactions are neglected. While semilocal DFT functionals similarly miss long-range correlations such as dispersion,^19^ purely local MLIPs also omit long-range electrostatics. For the second requirement, there is a wider array of techniques. For example, early work in the mid-1990s and early 2000s employed carefully chosen coordinate systems that respect the system’s symmetries.^20–22^

A shortcoming of this approach has been that it could not be easily applied to different systems. This limitation has been mitigated by the introduction of descriptors, which are representations of the local atomic environment that respect the necessary symmetries.^15^ One prominent class of such descriptors is the so-called atom-centered symmetry functions (ACSF).^15,23^ Although many different types of descriptors exist, including ACSF-based descriptors,^15,23–26^ the smooth overlap of atomic positions (SOAP),^27^ the spectral neighbor analysis potential (SNAP), moment tensor potentials,^28^ and the atomic cluster expansion (ACE),^29^ they share the common goal of encoding the local atomic environment in a symmetry-respecting way.

To make this concrete, consider the high-dimensional neural network potential (HDNNP) of Behler and Parrinello,^15^ in which each atomic energy *E*_*i*_ (eq 1) is predicted by a separate neural network NN taking a descriptor vector **G**_*i*_ as input,

2 $$E_{i}=\mathrm{NN}\left(G_{i};\theta \right),G_{i}=\left[G_{i}^{\left(1\right)},G_{i}^{\left(2\right)},...\right]$$

where NN­(·; **θ**) denotes a neural network with learnable parameters **θ**. A typical radial symmetry function that encodes the neighbor distribution around the atom *i* is

3 $$G_{i}^{\left(1\right)}=\sum_{j\neq i}e^{-\eta {\left(∥r_{\mathrm{ij}}∥-\mu \right)}^{2}}f_{c}\left(∥r_{\mathrm{ij}}∥\right)$$

where ∥**r**_*ij*_∥ is the distance between the atoms *i* and *j*, η and μ are hyperparameters controlling the width and center of the Gaussian, and *f*_c_ is a smooth cutoff function making sure that contributions vanish continuously at *r*_c_. To capture angular information, the radial functions are supplemented with angular functions:

4 $$G_{i}^{\left(2\right)}=2^{1-\zeta }\sum_{j,k\neq i}{\left(1+\lambda \;\cos\left(\theta_{\mathrm{ijk}}\right)\right)}^{\zeta }e^{-\eta ({∥r_{\mathrm{ij}}∥}^{2}+{∥r_{\mathrm{ik}}∥}^{2}+{∥r_{\mathrm{jk}}∥}^{2})}f_{c}\left(∥r_{\mathrm{ij}}∥\right)f_{c}\left(∥r_{\mathrm{ik}}∥\right)f_{c}\left(∥r_{\mathrm{jk}}∥\right)$$

where θ_*ijk*_ is the angle between **r**_*ij*_ and **r**_*ik*_, λ ∈ {−1, 1}, η and ζ are hyperparameters that control the spatial and angular sensitivity of the function to the local atomic environment, and *f*_c_ is again a smooth cutoff function. By construction, **G**_*i*_ is invariant under translation, rotation, and permutation of same-element atoms, so eq 2 satisfies all required symmetries.

Instead of using manually designed descriptors, one may learn descriptions of the local environment from available training data. In this approach, the system is encoded as a graph, with atoms as nodes and edges connecting all nodes within a cutoff distance. This graph is then used as input for a graph neural network.^30^ Each atom is initially assigned a feature vector, either derived from atomic properties or randomly initialized, that serves as a numerical representation of the atom within the system. During training, atoms connected by an edge exchange their feature vectors, a process usually called “message passing,” and then update their own features based on the received features^31,32^ (see Figure 1 for an illustration of this process).

**Figure 1:**
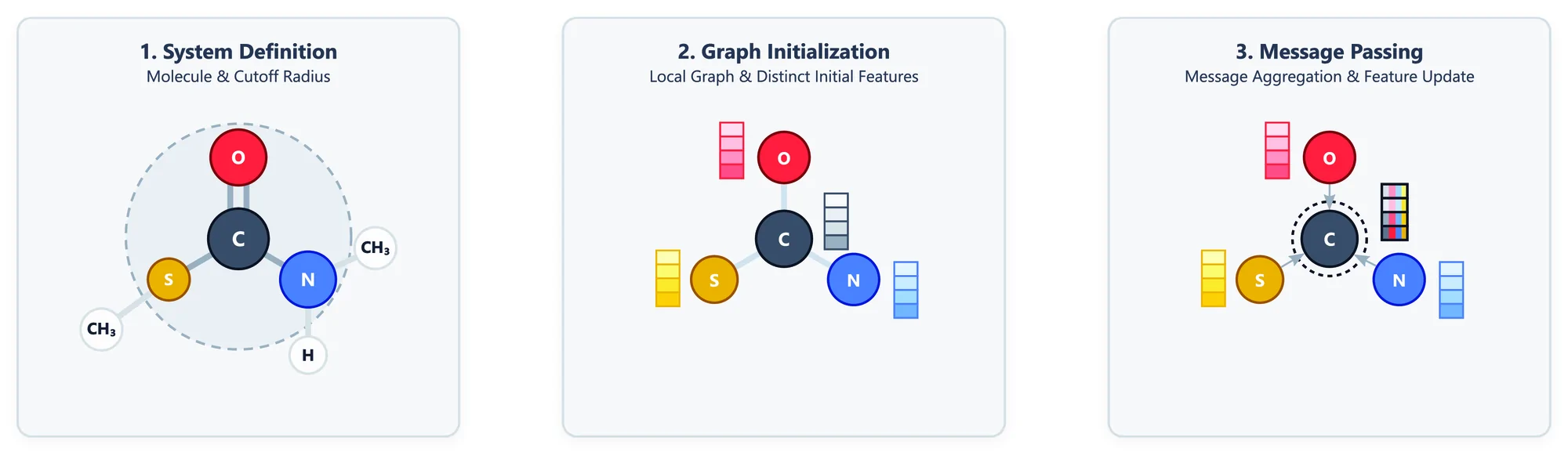
Illustration of the message passing scheme applied to a molecule. (1) A local environment is defined for each atom using a cutoff radius. (2) The resulting graph is constructed, and each atom is assigned an initial feature vector. (3) Each atom passes its feature vector to its neighbors. An atom then uses its own feature vector and those gathered from its neighbors to update its representation. For clarity, only the update of the carbon atom is shown.

Initially, graph-based models used features, such as distances and angles, that were invariant with respect to the symmetries of the systems.^33–35^ Since energy remains invariant under rotation, translation, and permutation of identical atoms, this approach does not present challenges for energy predictions. However, tensorial properties, such as forces, change orientation under rotation of the system, making predictions based on invariant features impossible. Models that use invariant features avoid this issue by calculating the force experienced by an atom as the negative gradient of energy with respect to the atom’s position. Similarly, other tensorial properties such as dipole moments or polarizability tensors can be obtained by predicting an auxiliary scalar function and combining its derivatives across atom pairs in a suitable tensorial construction.^36^

This limitation is addressed by equivariant models, which are models whose features transform in the same way as the input.^37,38^ Many of today’s state-of-the-art equivariant models are constructed using features based on spherical tensors represented by spherical harmonics.^17,39–44^ To maintain equivariance when aggregating tensorial features, the Clebsch-Gordan tensor product is used, but computing it, which is required during training and inference, is computationally expensive. An alternative line of work uses Cartesian tensors instead, which is conceptually simpler and computationally less demanding. However, most architectures are restricted to tensors of rank 1 ^45–47^ or rank 2,^48^ and extensions to higher-order tensors are an exception.^49–51^

In the low-data regime, (rotationally) equivariant models have been empirically found to improve data efficiency, meaning that fewer training samples are required to achieve the same level of accuracy.^39^ However, enforcing rotational equivariance often comes at the cost of more complicated architectures and slower models. This motivated research into alternative approaches such as adding penalty terms to the training procedure, which discourage violations of symmetry during optimization and thus guide the model to learn the necessary symmetries,^52^ and using data augmentation to learn symmetries from data.^53^ Recently, models based on the transformer architecture, such as PET, EScAIP, and AllScAIP, demonstrate competitive or state-of-the-art performance with a higher computational efficiency, typically by relaxing rotational equivariance while retaining other physical priors such as locality.^54–56^ Other studies, such as ref 57, go even further by abandoning the graph structure and thus learning locality from the data as well. Whether or not architectures with relaxed equivariance constraints will ultimately outperform equivariant ones at scale remains an open question. A systematic scaling study found equivariant models to outperform nonequivariant ones at every compute budget tested, but this result assumes comparable floating-point operations throughput between implementations, which may not hold in practice, because nonequivariant architectures can be easier optimized for high hardware utilization.^58^

## 3 The Breakthrough: Foundation MLIPs

The term “foundation model” has its origins in the field of natural language processing, where large models such as BERT were trained on large amounts of text and then achieved state-of-the-art results on a wide variety of tasks, such as question answering and natural language inference (i.e., determining logical relationships between sentences, such as whether one statement entails or contradicts another), through fine-tuning.^59,60^ This approach marked a paradigm shift: models are trained on large and diverse data sets and then fine-tuned for specific tasks. Scaling up both the model and the training data resulted in emergent capabilities for which the models were not explicitly trained,^59^ which greatly contribute to their success and their increasing popularity. While the term “universal MLIP” was used initially, the term “foundation model” has since been adopted in the field of MLIPs. However, it should be noted that while the early “universal” models have broad elemental coverage, for many systems they still required fine-tuning. Whether the criteria of predictable scalability and emergent behavior, as observed in the field of natural language processing, have been demonstrated for MLIPs remains an open debate.^61^ While we adopt the term “foundation model” in this Perspective for consistency with common usage in the field, we want to emphasize that we are referring to large, pretrained MLIPs, without insisting on predictable scaling or emergent behavior. In the realm of MLIPs, the initial focus was on training models for specific chemical systems, limited to certain elements and geometrical arrangements. However, with the advent of foundation MLIPs, there has been a shift toward applying large, general models that do not require extensive system-specific training.

The first generation of foundation MLIPs demonstrated that general-purpose interatomic potentials can be achieved in principle. The Materials Graph Network (MEGNet) was trained on data from approximately 60,000 crystals in the Materials Project database, covering 89 elements of the periodic table.^62,63^ It showed that a single model could deliver low prediction errors for a wide range of properties. Since then, a plethora of new foundation MLIPs trained on large data sets have been published, including M3GNet, MACE-MP-0, and eqV2, for example.^16,35,64^

These models have then advanced in several ways: they have grown in parameter size, adopted newer architectures, and trained on larger data sets. While the original MEGNet model had approximately 161 000 trainable parameters, M3GNet had 2,28,000; MACE-MP-0 had 4.7 million; and eqV2-L had about 153.8 million. Similar trends can be observed in the sizes of the training sets used. These advances have brought about a paradigm shift in the field, enabling stable molecular dynamics, high-throughput material discovery, and property predictions across a vast chemical space, all without the need for retraining.^35^ However, achieving quantitative accuracy for specific applications may still require fine-tuning, as discussed in Section 3.1.

Furthermore, MACE-MP-0, trained primarily on the MPtrj data set with a strong materials focus, showed encouraging out-of-distribution transferability, including qualitatively stable MD of liquid water.^16,65^

Although these models deliver good results on many systems, the training data used ultimately place constraints on their use. For example, systematic underestimation of energies and forces was observed in M3GNet and MACE-MP-0, and since the models were largely trained on DFT data from bulk materials, they have difficulty predicting surface energies.^66,67^ Another study found that predicting the adsorption energies of different N_*x*_H_*y*_ species on Ni(111), Ru(0001), Ru­(101 0), and Ru­(101 5) requires additional fine-tuning for all tested models, including eqV2.^68^

Further progress in addressing the limitations imposed by the training data used came with the Universal Model for Atoms (UMA), a model trained on a data set of half a billion structures.^17^ Not only is this data set of previously unattained size, but it also spans a wide range of domain-specific areas, including materials, molecules, catalysis, molecular crystals, and metal–organic frameworks. For this purpose, the five data sets OMat24, OMol25, OC20, OMC25, and ODAC25 were combined, and a single model was trained on all of them.^64,69–73^ Note that the newest version of UMA evaluated in this work (UMA-S-1.2) was also trained on the catalysis data sets OC22 and OC25, as well as an expanded molecular training set OMol25 and the newly released OPoly26 polymer data set.^74–76^ While training is usually performed with data generated using a single exchange-correlation density functional as the DFT reference, for the UMA model, training was performed on a combined data set generated using different density functionals. This was made possible by introducing the ’task’ label (referring to a chemical domain and to the reference method used for producing the corresponding training data set) as additional input to the model. This allows the model to build shared representations while still predicting quantities that depend on the task. At inference time, at least currently, the user must specify which task to use for a prediction, which might be challenging for systems that cannot be mapped cleanly to one of the tasks the model was trained on. Analysis showed that knowledge transfer occurred across the data sets, with a model trained on all data sets outperforming one trained exclusively on molecular crystal data.^17^ Of particular importance for UMA’s molecular chemistry capabilities is its training on the OMol25 data set. It is the first large-scale data set with broad chemical diversity across the periodic table, including various charge and spin states, computed with a modern exchange-correlation functional.^69^ While there are three UMA model variants, ranging from 150 million to 1.4 billion parameters, even the smallest variant was shown to achieve results generally comparable to, or better than, specialized models across a broad range of domains without any fine-tuning.^17^ This has since been corroborated. For instance, the smallest UMA model variant was applied to transition-metal complexes without undergoing additional training. The results showed that reliable conformer ranking is possible for most ligands in rigid systems but is less reliable for flexible or fluxional systems, where energy differences fall below a few kJ/mol and thus approach the accuracy of DFT itself.^77^

This development constitutes a paradigm shift within the field of MLIPs, demonstrating that large, general models can be applied throughout many domains, much like foundation models in natural language processing. Several large models trained on big data sets have now been released, differing in the chemical domains and configurational space covered by their training data, which, to a large extent, determines where they can be applied.^17,43,44,53,56,64,65,78–83^ For instance, MACE-MP-0’s material-focused training (see above) implies that it cannot be used interchangeably with models trained on broad molecular chemistry data sets such as OMol25. Additionally, the different architectures and model sizes lead to different trade-offs in terms of accuracy and speed.

Early applications demonstrate the versatility of these models. For example, MACE-MP-0 has been shown, without system-specific retraining, to accurately simulate the metastability of siliceous zeolites relative to α-quartz and the phase transition pressures of silica polymorphs under high pressure, with results aligning well with experimental values.^84^ Similarly, a systematic comparison of approximately 10,000 semiconductors indicates that MatterSim-v1 accurately predicts harmonic phonon properties, with errors smaller than the difference between the exchange-correlation functionals PBE and PBEsol, suggesting that some foundation models have achieved DFT-level fidelity in vibrational properties.^85^

### 3.1 Static Generalist Models vs (Repeated) System-Focused Fine-Tuning of Foundation MLIPs

As discussed in the previous section, foundation models have arrived in chemistry, and they are here to stay and make a lasting impact. However, the question now arises as to how these models should be applied. Should they be used as static, generally applicable models or should they be fine-tuned to specific systems? We structure the comparison along four decision axes that directly influence this choice: (1) accuracy relative to application tolerances, (2) maintenance risk due to catastrophic forgetting under iterative updates, (3) data and computing constraints, and (4) the need for reliable uncertainty estimates.

#### 3.1.1 Accuracy

As mentioned above, foundation models often achieve an accuracy level that is equivalent to or better than that of system-specific models. However, ultimately, it is important to assess the accuracy on a case-by-case basis against application tolerances. For instance, the predictions for diatomic molecules outside equilibrium bonding have been found to be inaccurate for some foundation models.^86^ Although certain models have addressed this issue by including diatomic data in the training set,^53,87^ this failure illustrates that accuracy must be assessed critically against application-specific needs and tolerances. However, if a static generalist model meets the target error thresholds in the relevant chemical areas, then using a generalist model can considerably reduce the required effort.

#### 3.1.2 Catastrophic Forgetting

If a generalist model cannot achieve the desired accuracy, an alternative is to fine-tune it to incorporate new knowledge. However, this increases the risk of “catastrophic forgetting”, i.e., a deterioration in performance in areas previously mastered.^88^ If the model is to remain generally applicable yet be expanded to include specific knowledge, catastrophic forgetting must be prevented while simultaneously keeping it flexible enough to incorporate new information. This tension is known as the stability-plasticity dilemma,^89^ addressed in the field of continual learning, for which many strategies have been proposed in the literature.

These strategies can be categorized as (i) regularization-based, (ii) replay-based, and (iii) parameter isolation.^90^ Regularization-based approaches add penalties or constraints to the loss function (the objective that is minimized during training) to prevent modification of important weights associated with learned knowledge,^91,92^ or to constrain the model’s predictions on previously learned data.^93^ Replay-based approaches periodically retrain on past training data to prevent forgetting.^94,95^ Parameter isolation approaches dedicate separate subsets of model parameters to each task, preventing interference between old and new knowledge.^96,97^

Most continual learning strategies have been developed for traditional machine learning tasks, such as image classification, and have only recently been applied to MLIPs.^26,98^ The most straightforward approach, and the one most frequently encountered in the MLIP literature, is to freeze large portions of the network and only adjust the weights in the final layers.^99–101^ Although this prevents catastrophic forgetting in the frozen layers, it significantly limits the ability to incorporate new knowledge.

A more targeted alternative strategy, called low-rank adaptation (LoRA), has been developed for large language models.^102^ Instead of adjusting the weights directly, they are frozen, and small, trainable, low-rank matrices are introduced. This enables structured, low-rank updates across all layers while preserving pretrained weights. This approach provides a more flexible balance between stability and plasticity than layer freezing. Although applying LoRA directly to equivariant MLIPs would break their equivariance, recent work has extended the concept to work with equivariant MLIPs.^103^ However, the application of continual learning methods to foundation MLIPs is still in its early stages, and their ability to balance stability and plasticity in practice has not yet been systematically evaluated.

#### 3.1.3 Data and Compute Cost

Static generalist models are ideal for large systems where generating training data would be computationally prohibitive. However, compared to system-specific models, training time is traded for longer inference times as the larger model size results in slower prediction speeds. In applications such as molecular dynamics, where chemical diversity is limited and inference time is critical, knowledge distillation is a promising approach. The goal is to transfer knowledge from a teacher (a static generalist) to a student model with fewer parameters. In doing so, general applicability is traded for significantly faster inference while maintaining, or in some cases exceeding, the teacher’s accuracy within the target chemical domain.^104–106^ This points to an emerging paradigm for MLIP development, in which published foundation models are used to create smaller, system-specific MLIPs that enable fast sampling without the need for additional training data.

#### 3.1.4 Uncertainty Quantification

Having an uncertainty estimate for predictions is important for many applications. For example, many active learning workflows use uncertainty to add new structures to the training data set.^107–111^ The most common approach is to use an ensemble of models, either trained on different data splits or with different initializations, and use the empirical variance calculated from individual predictions.^112–114^

However, in practice, a single static generalist model is typically released, which makes ensemble-based uncertainty estimation inapplicable in this context. Although there are other techniques, such as Monte Carlo dropout,^115,116^ mean-variance estimation,^117^ and loss trajectory analysis for uncertainty measures,^118^ these generally need to be integrated during model training. By contrast, system-oriented fine-tuning allows for the inclusion of additional (lightweight) prediction heads and the subsequent use of ensemble methods.^119,120^ However, the quality of these uncertainties must be carefully evaluated. For example, it has been reported that uncertainties can be significantly underestimated.^120^ When uncertainties must be estimated for a single static generalist without fine-tuning, several post hoc approaches are available: For example, gradient information can be used to measure the sensitivity of predictions as an approximation of uncertainty.^121,122^ Alternatively, distance-based methods flag structures as uncertain if they are far from the training data in the model’s learned feature space. These methods do not modify the model and only require a set of reference calculations for calibration.^123,124^ While gradient-based methods only need access to the training data once to precompute some quantities, distance-based methods still need access to the training data during inference. However, even a single iteration through the training data can be computationally demanding if the data comprise millions of structures.

Gradient-based uncertainty methods are the natural first choice for a static generalist model, because they do not require model training or access to training data during inference. However, there must be sufficient computational power to iterate through the entire training data set once. To validate the uncertainty estimates and assess the accuracy of the generalist model for the target system, a small set of reference calculations is, nevertheless, recommended. If the model’s predictions are not accurate enough, then fine-tuning can be attempted using the reference calculations generated as training data.

### 3.2 Performance of MLIPs

Comparing the computational cost of *ab initio* methods and classical force fields with that of MLIPs is not as straightforward as one might think. First, these conceptually different approaches have different hardware requirements. For instance, many traditional DFT programs are optimized primarily for central processing units (CPUs), whereas MLIPs perform best on graphical processing units (GPUs). However, we note that there are several DFT programs available (e.g., TeraChem, QUICK, GPU4PySCF) that offer GPU acceleration.^125–128^ Second, it is not clear which measure is most suitable for the comparison: Should the focus be on runtime or on the computing power measured, for instance, in floating-point operations per second? What about power consumption, which can vary greatly between CPUs and GPUs? In addition, what degree of accuracy and system size should be compared? Since these methods have different time complexities, changes in system size can drastically alter the results. Given these difficulties and the fact that exact runtime differences depend on the specific application, our goal here is not to provide exact numbers. Instead, we intend to provide guiding estimates. A more comprehensive benchmarking would need to consider the accuracy of energies and forces, as well as the suitability of MLIPs for downstream tasks such as structure optimization or stability in molecular dynamics simulations. For such benchmarks, we refer the reader to other work, such as MLIP Arena and LAMBench.^129,130^

To understand how MLIPs perform compared to classical force fields and DFT, we performed single-point calculations (i.e., energy and force evaluations) on a naphthalene structure from the MD17 dataset^131^ and on a paclitaxel structure. For DFT single-point calculations, we used the NWChem interface of the Atomic Simulation Environment (ASE) with the PBE0 exchangecorrelation functional and the def2-SVP basis set.^132–135^ To allow for comparisons with classical force fields, we performed single-point calculations on the same structures using the universal force field (UFF) in Open Babel.^136,137^

The measured timings are shown in Table 1. We emphasize that their main purpose is for illustration rather than for providing a rigorous benchmark, which would require significantly more work that is beyond the scope and idea of this work. Still, one can easily see that the overall conclusion is that force fields are roughly 40 to 40 000 times faster than the MLIPs studied, whereas DFT calculations can be anywhere from roughly 80 times up to nearly 900 000 times slower than MLIPs, depending on system size, hardware, and the chosen model.

**Table 1:** Comparison of Runtimes in Milliseconds of a Single-Point Calculation (i.e., Energy and Force Evaluation) for Naphthalene and Paclitaxel on an Intel Core i7-14,700K Processor (4 Cores Columns) and an NVIDIA GeForce RTX 4090 Graphics Card (GPU Columns)^a^

	naphthalene		paclitaxel	
method	4 cores	GPU	4 cores	GPU
MACE-MH-1 (omol)^44^	56.463	34.650	549.281	36.10
GRACE-2L-OAM^83^	7.41	4.42	21.96	9.37
MatterSim v1 5 M^78^	21.00	25.24	225.37	30.40
eqV2-M^64^	366.28	58.80	2830.68	108.25
Orb-v3 (omol)^79^	37.84	9.99	500.49	14.00
SevenNet-MF-ompa (omat24)^139^	97.98	38.94	2021.57	39.82
UMA-S-1p2 (omol)^17,87^	111.46	48.70	951.23	48.02
Nequix-MP-1 ^81^	4.68	2.89	42.57	4.67
PET-MAD-1.5-S^53^	27.93	23.73	180.31	23.77
UFF^136^	0.01		0.11	
PBE0 ^134^	30533.94		4137626.60	

a Where applicable, the used output head or model variant is specified in parentheses.

When we compare the time required for a single-point calculation across different MLIPs, significant differences in performance become apparent. For example, on the CPU, eqV2-M is about 66 times slower than Nequix for paclitaxel and about 78 times slower for naphthalene. However, this is to be expected, since Nequix has around 700 thousand parameters, while eqV2-M has 86 million. It is also evident that the models do not all benefit equally from using a GPU (right column in Table 1). On a GPU (compared to the CPU), on the larger system, eqV2-M is more than 25 times faster, while GRACE-2L-OAM is only about twice as fast on the GPU. However, on average, the MLIPs are more than five times faster on a GPU compared to the CPU (geometric mean of the speedup ratios: 5.28-fold), and for every model tested, the speedup is greater for the larger system (paclitaxel).

We note again that the provided timings are not intended for direct comparison between MLIPs, as they differ in parameter counts, accuracy levels, and the data set they were trained on. Moreover, timings are affected by the system size, leading to different relative timings in larger systems. Our main objective here is to provide a general idea of how MLIPs compare with force fields and DFT.

Our comparison of MLIP timings with those of the UFF further supports the existing literature that indicates that classical force fields are considerably faster (between approximately 40 and 40 000 times depending on the model, system and hardware).^138^ Similar substantial time differences can be observed between DFT and MLIP results, with the MLIP being orders of magnitude faster. The comparison between naphthalene and paclitaxel further shows that the advantage increases as the system size grows.

While the drastic speed difference between classical force fields and MLIPs might seem to favor strongly the former, it is important to emphasize that the speedup is achieved by using a highly efficient but narrow and system-specific parameterization. MLIPs, on the other hand, are trained on broad, first-principles-derived data, allow for systematic improvements by increasing the training data coverage, and generalize well beyond a single, narrowly defined system. Hence, MLIPs should not be seen as a replacement for force fields (where available) but rather as taking over the role of DFT (or even more accurate quantum chemical approaches in the future) where force fields are unavailable or not accurate enough.

## 4 Challenges Pointing to Future Developments

Despite their impressive performance, foundation models for atomistic simulations still face several unresolved challenges. These challenges include limitations in the description of systems, computational scalability, data quality, and reliability. Overcoming these challenges will be crucial to realizing the full potential of MLIPs for real-world applications.

### 4.1 Physical Fidelity

A significant issue is their dependence on a fixed cutoff radius, typically ranging from 4.5 to 10 Å. Although message passing enables models to use information beyond this cutoff, information diffusion is ultimately limited by the cutoff and the number of message-passing steps. This restricts the inclusion of long-range electrostatics, induction, and dispersion interactions,^140^ which are crucial, however, in the simulation of large atomistic assemblies such as metal–organic frameworks and biomacromolecules.^141,142^

Long-range electrostatics and induction are, up to approximations in the exchange-correlation functional, captured in the DFT formalism, and thus part of the training data. The issue for MLIPs is that configurations that have the same local environment but different global configurations can have different energies. Various studies have addressed this issue, for example, by predicting partial charges or Wannier centers and summing the resulting electrostatic interactions,^34,143,144^ or by building nonlocal descriptors that can target both electrostatics and dispersion.^145,146^ An alternative to explicit charge prediction is the Latent Ewald Summation (LES) method, which instead infers latent charges purely from total energies and forces.^147^ This removes the need for explicit charge labels in the training data, making LES compatible with the majority of published data sets. Other approaches use charge equilibration (Qeq) schemes,^148^ in which electronegativities and hardness are predicted from local geometry^136–138^ and then used to minimize a quadratic energy functional with respect to atomic charges subject to the constraint of a fixed total charge. This enables the model to capture long-range charge transfer and redistribution across the system, but increases the computational overhead. We note that methods that determine charges globally capture a degree of induction alongside electrostatics by design.^149^

Although most foundation models currently available do not directly account for long-range effects, the recently published models MACE-POLAR-1 and AllScAIP already do so, using two contrasting strategies.^56,150^ MACE-POLAR-1 uses a physics-driven approach that extends the MACE architecture by including explicit long-range interactions. Atomic multipoles are iteratively updated by the local electric field using a nonself-consistent field formalism. This is followed by global charge and spin equilibration, which is enforced via learnable Fukui functions.^150,151^ AllScAIP, on the contrary, relies on a data-driven strategy. It combines a local neighborhood attention step with a distance-based cutoff and an all-to-all node attention step with no cutoff. This enables the model to learn long-range interactions from data.^56^

Unlike long-range electrostatics and induction, dispersion interactions, caused by instantaneous electron density fluctuations, are absent from semilocal DFT functionals. In DFT, this is often corrected using empirical corrections such as D3.^152^ While an MLIP can be trained directly on energies that already include such a correction, the fixed cutoff of the model means that, just as for electrostatics and induction, the model cannot distinguish between configurations with identical local environments but different global arrangements, and therefore always predicts identical energies for them. Alternatively, empirical dispersion corrections can be applied to MLIPs at inference time, independent of the cutoff, as done, for example, in PhysNet,^34^ which augments the model with D3 correction. A simpler approach augments the MLIP with explicit *r*_*ij*_^–6^ terms.^153,154^ Other approaches predict the inputs to a many-body dispersion (MBD)^155^ correction directly from the local environment, such as Hirshfeld scaling ratios^156^ or atomic polarizabilities and *C*_6_ coefficients also used in MBD.^157^

It is important to note that incorporating information beyond a local cutoff means that the computational complexity of the model no longer scales strictly linearly with the number of atoms, ranging from O(Nlog⁡N) for Ewald-based methods (which can be accelerated further with fast multipole approaches) up to $O\left(N^{2}\right)$ for AllScAIP and $O\left(N^{3}\right)$ for a naive implementation of MBD.^157^

Regarding future developments, the jury is still out on the best approach to incorporating long-range effects into foundation models. Should the known physical form of long-range interactions be encoded explicitly or should sufficiently flexible architectures be used to learn the functional form of long-range interactions directly from data? The latter might have advantages, as it could capture such effects in a very general sense (not bound to, and therefore beyond, analytic dispersion expressions), but accurate reference data for training would be difficult to generate.

Another limitation is the neglect of the total charge and spin multiplicity in many MLIPs.^16,64,78,81,83,139^ While incorporating these parameters as additional global input values is straightforward at the architectural level, as already done by some models,^17,44,79^ training such models requires data covering a wide range of charge and spin states. However, spin multiplicity alone is insufficient for many magnetic phenomena. Simulating antiferromagnetism, for example, requires explicit information about local magnetic moments. Unlike total charge and spin multiplicity, local magnetic moments are per-atom quantities that cannot simply be appended to the global input, and in the case of noncollinear magnetism, those are vectorial quantities that need to be integrated into the message-passing scheme. Several works have addressed the construction of magnetic MLIPs that incorporate local spin degrees of freedom,^158–162^ although these are system-specific models rather than foundation models. A first step in this direction was taken with the CHGNet model,^65^ which predicts collinear magnetic moments. However, the magnetic moments are not input parameters in this model, and therefore, the model cannot distinguish between structures with the same geometry but different spin configurations. Although recent foundation models have advanced in incorporating total charge and spin multiplicity as global inputs, incorporating local magnetic moments and extrapolating to unseen spin configurations and charge states remains a challenge requiring both further architectural development and expanded training data.

Foundation models, as discussed here, have been restricted to the ground-state potential energy surface so far, and therefore, they cannot be used directly for excited-state and photochemical processes (yet). While several studies address excited-state and nonadiabatic dynamics with related ML architectures (see, for instance, refs 163,164 ), integrating this capability into foundation models remains an open and feasible direction for future work.

### 4.2 Scalability and Efficiency

MLIPs are usually trained using energy and forces. However, many applications, such as transition-state (TS) searches, require Hessians. For models in which the force is predicted rather than calculated as a gradient of the energy, incorporating Hessian information during training has been shown to be crucial for successful TS searches.^165^ Although incorporating Hessian information can also improve the performance in TS searches of models in which the force is computed as the gradient of the energy,^165,166^ there is also evidence that a model solely trained on energies and forces can, via automatic differentiation, produce Hessians accurate enough to be used successfully in TS searches.^167^ Furthermore, incorporating Hessians into the training process has also been shown to improve vibrational frequency predictions.^168^ However, these findings were demonstrated on data sets several orders of magnitude smaller than those used to train current foundation models. Generating reference Hessian models using DFT at scale remains computationally expensive, and training MLIPs with Hessian supervision also greatly increases the computational cost of training. Recent work has already addressed this by training against stochastic projections of the Hessian along random directions, rather than explicitly constructing the full Hessian matrix,^169^ and by pretraining on Hessians calculated with a cheaper method, combined with targeted inclusion of Hessians calculated with a more accurate method.^170^ Whether Hessian-informed training is beneficial as data sets grow larger and whether these strategies are sufficient to make it computationally practical at scale, remain open questions.

Although comparisons of MLIPs are often limited to energy and force errors, it has been shown that models with small force errors can still fail in molecular dynamics simulations.^171^ Therefore, benchmarks that more closely resemble actual applications are essential. One notable benchmark that contributes to this effort is the MLIP Arena.^129^ Looking ahead, it will be important to develop more advanced training paradigms that not only utilize energies and forces but also adapt models directly to specific tasks such as molecular dynamics simulations. A currently underexplored dimension for comparing MLIPs is fine-tuning efficiency, that is, the amount of data required to reach the target accuracy in a specific chemical domain. Such a benchmark would aid model selection in areas where current models are not accurate enough, and fine-tuning is needed.

### 4.3 Data and Evaluation

The accuracy of MLIPs depends on the training data set. The current approach to building foundation models is to train them on increasingly large data sets that span a wide variety of chemical systems. However, constructing such data sets is difficult and presents several challenges. Not only is it important to sample diverse chemical domains, but the configurations sampled within each domain are of great importance as well. Models trained purely on data sets based mainly on near-equilibrium structures will perform poorly in reactive simulations, due to an undersampling of high-energy or high-force structures. Dedicated sampling strategies, such as algorithms for automated exploration of reactive regions of the potential energy surface,^172–175^ and active learning from machine learning-driven molecular dynamics,^176^ are needed to build data sets that support such applications.

At the same time, quantities relevant to reactive chemistry, such as barrier heights, have strict requirements for accuracy. Since these enter rate equations in the exponential, even small changes can lead to drastic effects in calculated rate constants. References 177 and 178 showed that models trained on OMol25 can handle reactive simulations (to some extent) out of the box. Furthermore, ref 76 showed that additional reactive polymer data was needed for accurate reactive simulations in polymeric environments, while ref 179 showed that for accurate reactive surface configurations, some fine-tuning is needed.

Beyond data set composition, quality control protocols are required to ensure that problematic data such as unconverged calculations or calculations troubled by numerical noise are filtered out to prevent such erroneous data from propagating to the trained model.^180^ For instance, a recent study revealed that several data sets contained structures with nonzero net forces, suggesting numerical noise in the DFT calculation and suboptimal DFT settings.^181^

Moreover, even correctly computed data can be problematic if they contain extreme outliers. Reference 79 showed that structures with unusually high energy, force, or stress values produced spurious oscillations in diatomic dissociation curves and that this could be improved by removing such outliers.

Besides the difficulties of sampling and curating data, training with ever-increasing amounts of data is computationally expensive. Without a clearer understanding of which data are important for training, a great deal of computing power could potentially be wasted.

Although the one-time cost of training is arguably less important than inference speed, keeping data set sizes manageable remains valuable. It allows smaller (academic) groups to experiment with training state-of-the-art models themselves without having to rely on a small number of organizations with a large-scale compute budget. Future work should therefore address both directions simultaneously: constructing manageable, well-curated data sets, and developing architectures for fast inference.

Current foundational models largely lack an integrated mechanism for quantifying uncertainty. Although there are numerous methods for measuring prediction uncertainty (see Section 3.1.4), most models are published without an integrated uncertainty estimation method. As a consequence, one must resort to post hoc approaches, such as readout ensembling.^120^ PET-MAD is a notable exception as a foundation model that has a directly integrated uncertainty estimation.^53^ However, foundation models must be equipped with well-calibrated uncertainty estimates to be practical for use. At the same time, developing uncertainty quantification methods that are both mathematically rigorous and computationally efficient remains an important open challenge for the field.^9,182,183^

To ensure that quantum chemical calculations are not necessary for routine and high-throughput computational campaigns, broad domain-specific studies are required to assess in which areas MLIPs are already reliable. Then, MLIPs can be used without resorting to expensive quantum mechanical methods, similar to how DFT is currently used without validating calculations with more accurate methods. In this sense, just as DFT users must understand which exchange-correlation functional and basis set to use for a particular application, MLIPs users must understand the training data underlying a model and thus its domain of applicability. But as we know from the use of DFT in practice, such knowledge is easily built up and spread in the computational chemistry and materials science community. In cases where access to the underlying electronic structures is required, such as for band gaps, models that predict the Hamiltonian^184–191^ or the one-body reduced density matrix are an attractive alternative to DFT.^192–194^ Even the electron density has been the target of successful machine learning approaches,^195^ providing just one example that electronic structure information can be learned reliably.

As a note to the reader, we would like to add that we are well aware of the rather drastic and consequential statements made in our Perspective. Their purpose is not a provocation; instead, they should serve as a wake-up call. As MLIPs are starting to replace DFT in routine calculations, it is important to be aware of the accuracy ceiling imposed by DFT on MLIPs. Traditional DFT applications are plagued by relying on a more or less semiempirical energy density functional that will always compromise accuracy to some (more or less known) degree, yielding results of, at times, questionable reliability, and that cannot be systematically improved. Therefore, method development needs to seriously consider the future of semiempirical exchange-correlation functional development compared to pushing the frontiers in wave function theory. Although these methods are currently too expensive to generate exhaustive training data, starting from a model trained on DFT data and refining it with a smaller set of more accurate *ab initio* data (an approach usually called transfer learning^196^) allows these accurate wave function-based methods to improve MLIPs, as shown, for example, in refs 101,197 .

## 5 Conclusion and Outlook

We are in the midst of a process that will fundamentally change chemistry as a scientific endeavor.^198^ Successes in machine learning require us to question how we do chemical research, what tools we use, and how and what we teach in chemistry.^199^ The efficiency and surprises of machine learning and artificial intelligence approaches have affected all branches of chemistry, also how we model chemical processes.

While theoretical chemistry has been based on the modeling of the explicit dynamics of atomistic entities (electrons, atomic nuclei, atoms, united atoms, etc.) deeply rooted in mechanical theories well established in physics, we now see a shift toward implicit models with surprising extrapolation capabilities emerging from data-driven approaches in the field now known as digital chemistry. Thomas Kuhn’s concept of a (fundamental) “paradigm shift”—unfortunately too often used in an inflationary manner—is actually seen here in action.

For the construction of potential energy surfaces, routine physics-based modeling will be replaced by implicit models that, in some impenetrable way, have inferred the physics from large data sets. Foundation MLIPs will become the starting point of computational chemistry campaigns, completely replacing DFT for this purpose. While their performance is already very impressive, future work along the lines of the open challenges discussed in this Perspective will further increase their reliability, accuracy, and general applicability. Accurate *ab initio* reference data will replace the DFT training data so that they can achieve (multireference) coupled cluster accuracy from the outset, beating DFT methods not only in terms of computational costs but also in terms of accuracy. Moreover, specific fine-tuning will always be possible thanks to rolling benchmarking calculations that can produce system-focused *ab initio* data in an automated way. Accordingly, accurate quantum chemical methods (obtained by traditional or quantum computation) will be used for generating training and validation data in routine computational chemistry.

While quantum chemical approaches have always come with the promise of rigor and systematic improvability (a desire, too often torpedoed by the curse of dimensionality in quantum mechanics), accurate error bars have remained inaccessible for some specific problems at hand in routine applications.^9^ By contrast, machine learning approaches offer several concrete pathways toward uncertainty quantification, such as ensembling, without direct analog in quantum chemistry. Realizing this potential will require addressing the issues discussed in Sections 3.1.4 and 4.3, namely integrating uncertainty estimation directly into foundation models and ensuring that predicted uncertainties reliably track true error. Once realized, this would allow for system-focused studies that do not require a well-defined underlying model. Instead of using acronyms for approximations (as in DFT), we will eventually resort to only error bars obtained from faithful uncertainty quantification procedures.

When the next-generation foundation MLIPs with automated fine-tuning and uncertainty quantification become available in standard software for computational chemistry, they will be tested by the community, and they will convince the community because of their easy applicability, accuracy, and speed, and because of the system sizes or number of systems that become computationally accessible. Akin to the DFT revolution in computational chemistry, we are now at the brink of the MLIP revolution, where MLIPs will become the new canonical approach to modeling chemistry. Thanks to user-friendly implementations of MLIPs, one might not even notice the shift from density functionals to MLIPs except that the calculations are then more accurate and faster. While the latter property is obvious, the former reference to accuracy anticipates that it will be only a matter of (short) time until we will have vast sets of accurate post-Hartree–Fock data available for training. Those will be based on standard coupled cluster theory in cases of weak electron correlation or on state-of-the-art multireference methods for strongly correlated electronic structures of molecules (either calculated by traditional means or, in the future, by quantum computation^200^). Compared with DFT, larger atomistic structures can already be handled now, and, in the case of molecular dynamics studies, longer time scales are accessible. MLIPs will then become more and more reliable by virtue of evolving training data sets of increased accuracy and further algorithmic and MLIP design improvements.

Accordingly, we may expect a whole new jargon to emerge in computational chemistry. But it will not only be the way we speak about computational chemistry simulations in routine applications that will change, but we can also even anticipate a whole new class of chemical concepts that will allow us to extract descriptive and predictive qualitative information out of MLIP calculations (as recently explored in ref 201), very much like standard chemical reactivity concepts that were rooted in quantum chemistry (especially within conceptual DFT).

MLIPs are also the data-driven solution to unite the fields of quantum chemistry and materials science (electronic structure) with polymer and (bio)­molecular simulations (force-field-based configurational screening): quantum accuracy of microstate energies will be so quickly accessible that enhanced sampling techniques can be exploited to represent configurational flexibility properly, but with quantum accuracy, a key to understanding all sorts of complex and functional processes in the molecular sciences.
